# Hearing impairment and its risk factors by newborn screening in north-western India

**DOI:** 10.1186/s40748-015-0018-1

**Published:** 2015-07-06

**Authors:** Zia Ul Haq Gouri, Deepak Sharma, Pramod Kumar Berwal, Aakash Pandita, Smita Pawar

**Affiliations:** Kothari Hospital and Research Center, Bikaner, Rajasthan India; Department of Pediatrics, Pt B.D. Sharma PGIMS, Rohtak, Haryana India; Department of Paediatrics, S.P. Medical College, Bikaner, Rajasthan India; Department of Pediatrics, Government Medical College, Jammu, India; Department of Obstetrics and Gynaecology, Fernandez Hospital, Hyderabad, India

**Keywords:** Transient evoked Otoacoustic emission (TEOAE), Auditory brain stem response audiometry (AABR), Newborn screening, Hearing impairment risk factor

## Abstract

**Background:**

To screen the newborn by Transient evoked Otoacoustic emission and to assess the incidence of hearing damage and associated risk factors.

**Method:**

This longitudinal prospective observational study was conducted at a tertiary care hospital in India. A total of 415 babies were included in the study. All the newborns were evaluated with Transient evoked Otoacoustic emission (TEOAE) which was done by age of 1–3 days. Auditory brain stem response audiometry (AABR) was performed at the age of three months for confirming the hearing loss in the neonates those who failed the TEOAE screening. For infants proven to have significant hearing loss in one or both ears, were denoted to an ear, nose, and throat specialist for further evaluation & rehabilitation.

**Results:**

Out of total 415 babies included in the study, 22 neonates showed abnormal TEOAE examination. Out of these 22 neonates, hearing loss was confirmed in 18 (82 %) subjects. by AABR. The following antenatal and post-natal risk factors were associated with hearing loss: ante-partum bleeding, history of maternal blood transfusion, fetal distress, prematurity, severe birth asphyxia, NICU admission for more than 24 h and Apgar score less than five at 5 min.

**Conclusion:**

Late identification of hearing loss presents a substantial public health burden. Early recognition and intervention prior to 6 months of age has a significant positive impact on development. A high incidence of hearing impairment seen in our study neonatal population warrants the urgent implementation of universal hearing screening of all the newborn infants in India. NICU infants admitted for more than 24 h are to have an auditory brainstem response (AABR) included as part of their screening so that neural hearing loss will not be missed.

## Background

Hearing impairment has a devastating, and detrimental impact on the development of newborn infants [1]. Neonates having bilateral hearing loss or unilateral hearing loss of varying degrees above 1000 Hz develop significant long term effects on speech and language sciences [2]. Reduced auditory input also adversely affects growth of the auditory nervous system, and can negatively affect the speech perception that interferes with the increment in social, emotional, behavioral, and cognitive spheres, academic achievement, vocational alternatives, employment, and economic self-sufficiency [3]. The major difficulty with late identification of hearing loss is the effect on language development. A delay in identification can mean a delay in establishing effective communication. Professionals agree that hearing loss in infants should be detected promptlyand appropriate audiologic rehabilitation should be instituted early, to take advantage of the plasticity of developing the sensory system (critical period is 0–3 years). This exploit can lead to normal speech and speech development, social, emotional, and cognitive development, and academic achievement in the youngster [4, 5]. In addition, identifying hearing loss before it is clinically apparent, provides a baseline on which subsequent evaluation can be developed and compared. Also, medical, and surgical treatment can be initiated for conductive hearing loss to limit its progression. Timely information also provides the acceptance of hearing damage and improves the parent’s readiness to begin a family centered rehabilitation program [6]. In many parts of the world routine newborn hearing screening has been implemented with varied success [7–13], but in developing world nations like ours universal screening is not available and has many obstacles to its implemenation [14, 15]. In India, there is no dedicated national program for early detection of hearing loss in newborns. Studies suggest that four out of every 1000 neonates have severe to profound hearing loss [16, 17].

The present study was designed to perform newborn hearing by Otoacoustic emission (TEOAE), determine the incidence of hearing damage in a population of at risk and not at risk neonates, and to determine various risk factor associations with learning impairment.

## Methods

We conducted a prospective observational longitudinal study in a tertiary care hospital in India, Department of Pediatrics, Sardar Patel Medical College, & Associated Group of Hospitals, Bikaner (Rajasthan). The study was approved by institutional research board (IRB) of the college.

During the 8 month study period, form March, 2011 to October 2011, a total 1125 neonates were born in our hospital. We decided to enroll 415 neonates in the study. All the neonates born during the study period were divided into neonates with and without risk factors using predetermined Joint Committee statement on infant hearing screening (JCIH) criteria. Then each neonate was given a sequential number and enrolled in the study using random numbers generated by the computer for each group and enrolled till our sample size reached 415.

### Inclusion criteria

All newborn babies born in our hospital from 3/11-10/11.

### Exclusion criteria

#### Fail to get parental consent

Out of total 248 newborns having no risk factor while 167 newborn had some risk factor for hearing loss as per American Joint Committee statement on infant hearing screening (JCIH) criteria [18]. The risk factors which were assessed included

1. Low birth weight (less than 2 lb) and/or prematurity.
2. Assisted ventilation (to aid with breathing for more than 10 days after parturition).
3. Low Apgar scores with severe birth asphyxia (defined as Apgar score of three or less at 1 min of age).
4. Severe jaundice after birth requiring exchange transfusion or serum bilirubin level >20 mg/decilitre.
5. Hydrocephalus
6. Maternal illness during pregnancy (for example, German measles [Rubella]).
7. An illness or condition requiring admission of 24 h or more to a NICU.
8. Stigmata or other findings associated with a known syndrome to include a sensorineural and/or conductive hearing loss.
9. Family history of permanent childhood sensorineural hearing loss.
10. Craniofacial anomalies including those with morphological abnormalities of the pinna & ear canal.
11. In utero infection by TORCH group of organisms.
12. Respiratory distress;(presence of at least two of the following criteria-respiratory rate more than 60 per minute/subcostal or intercostal recession/expiratory grunt or groaning).
13. Meningitis and sepsis with positive CSF and blood cultures respectively.
14. Parental concern.

Informed written consent was obtained from the parents of all babies. Thorough Ear, nose, and throat examination done before doing Transient evoked Otoacoustic emission (TEOAE) that includes looking at the morphological abnormalities of the external ear. These newborns were screened for hearing impairment using the following test protocols.

- Transient evoked Otoacoustic emission (TEOAE) was employed as the first stage of screening by the age of 1–3 days.
- Auditory brain stem response audiometry (AABR) was performed at the age of three months for confirming the hearing loss if the neonates failed the TEOAE screening.

Children who had normal AABR were declared as ecologically sound & no further evaluation was suggested to these children.

All neonates were screened with TEOAE testing in a quiet room adjacent to the NICU. Otoread TEOAE screeners (Interacoustic Ltd., Assens, Denmark) was used for testing. The TEOAE was done by an audiologist who was trained in doing the TEOAE and this test was done free of cost. The Quick screen mode was used with a specially designed “stop” protocol that forced discontinuation of the protocol when “pass” criteria was met. The timing window was 12.5 milliseconds, and clicks were delivered at a rate of 80 per second. Stimuli consisted of standard transient clicks at 70 to 88 dB pSPL.

Otoacoustic emissions were judged to be present and an ear to have “pass” when signal-to-noise ratio was at least 3 dB in at least three of four frequency bands (1000, 2000, 3000, and 4000 Hz). A minimum of 60 successful sweeps was achieved in the test to be considered valid. Screening continued until passing criteria were met or 1000 successful sweeps were occurring, or for 10 min. If a baby failed an initial screen, it was repeated immediately after an effort to troubleshoot i.e. improve probe fit, clean probe contaminated by dust, decrease ambient noise, or change site of testing, calming baby by swaddling, rocking, and eating. An acceptable screen was done but once for each ear.

Follow-up testing consisted of AABR testing that was performed under conditions of natural sleep using an Evomatic 4000 evoked potential unit (Medtronic, Minneapolis, MN), a standard Ag/AgCl electrode applied on the forehead and each mastoid, and pediatric insert earphones coupled to Etymotic ER3A stimulators (Etymotic Research, Elk Grove, IL). The AABR was done by a trained audiologist and was done free of cost. Stimuli consisted of 100-millisecond rarefaction clicks and tone pips presented at a rate of 25 per second for at least 1000 presentations with alternating triggering to permit both ears to be examined simultaneously. Our cutoff values of normal were 23 to 26 dB for observable wave V for clicks and 30 dB NHL for tone pips.

For babies who failed the initial TEOAE screening, the mother, & relatives were contacted in person and counselled as to the significance of a broken screen (i.e. suggested need for further evaluation, not diagnosis of hearing loss). These neonates’ parents were also contacted in between for giving them a reminder for AABR and on the day of examination, they were contacted in personnel to get the test done.

Auditory brain stem response audiometry was used to confirm the hearing loss if the neonates failed the TEOAE screening at the age of 3 months.

For infants proven to suffer substantial hearing loss in one or both ears, were referred to an Ear, nose, and throat specialist for further evaluation & rehabilitation.

### Statistical analysis

All the patient data were entered into Microsoft excel sheets. The Statistical Package for the Social Sciences (SPSS) software version 16 for Windows was used for data analysis. Student’s *t* test and Chi-square test were used for data analysis. A *p* value less than 0.05 was considered significant.

## Results

In the present study, we have performed the initial screening of 415 newborn by TEOAE, only 22 babies had abnormal results. These 22 babies were further evaluated by AABR at age of 3 months. AABR showed 18 babies with confirmed significant hearing loss. Out of the 22 babies with abnormal results, 16 babies were in with any risk factor group and remaining babies were in without any risk factor group.

The demographic data of the study population, including gestational age, birth weight, gender stratification in with, and without risk factor groups are summarized in Table 1. During the study period neonates with suspected sepsis or clinical sepsis received cefotaxime and amikacin as a first line empirical antibiotic therapy. The newborn with asphyxial encephalopathy with sepsis, nephrotoxic drugs were avoided. The antibiotics were discontinued within 48 h if cultures were sterile. The second line of antibiotics was piperacillin tazobactum or Cefepime and antibiotics were changed as per the sensitivity pattern of the microorganism. In neonates with renal failure drug dose adjustment was performed.

**Table 1:** Table showing demographics of the population -gestational age, birth weight, gender stratification in the with and without risk factor groups

Distribution of total study population according to association of risk factor		
	Without risk factor	With risk factor
Gestational age		
<30 weeks	8	10
30–33 6/7 weeks	8	20
34–36 6/7 weeks	32	29
≥37 weeks	200	108
Gender		
Male	137	93
Female	111	74
Birth weight		
<1.5 kg	7	8
1.5–2.49 kg	53	54
>2.5 kg	188	105

**Fig. 1:**
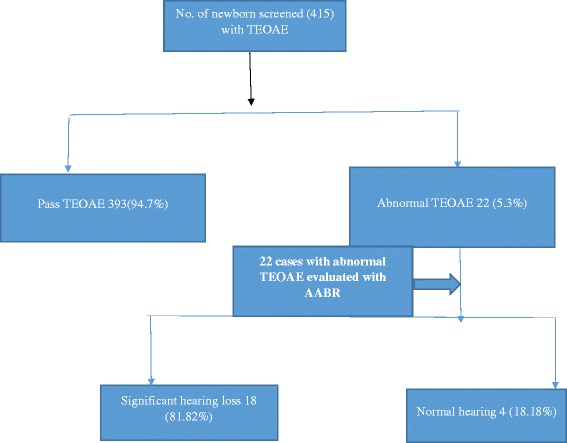
Flow diagram with distribution of cases screened by TEOAE and cases with abnormal TEOAE evaluated with AABR

Out of the 415 newborns screened with TEOAE test 22 (5.68 %) babies had abnormal screen either in single or both ears. Out of total 22 abnormal TEOAE cases, 18 (81.82 %) cases were taking in significant hearing loss with AABR while four (18.2 %) cases had normal AABR (Fig. 1).

**Fig. 2:**
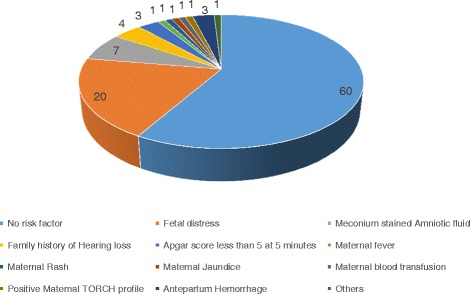
Pie diagram showing the risk factor stratification in the study population

The comparison of various risk factors between normal AABR and abnormal AABR shows that Apgar score at 5 min less than five, NICU admission, the presence of fetal distress, presence of meconium stained amniotic fluid are significantly important predictors for hearing loss (Table 2). The risk stratification of the various factors in the study population has been depicted in the pie diagram (Fig. 2).

**Table 2:** Table comparing various risk factors associated with hearing loss in BERA positive cases

	Normal hearing	Hearing loss	*P* value
Mean gestational age (weeks)	36.93 ± 2.41	36.71 ± 2.17	0.051
Birth weight (kg)	2.667 ± 0.50	2.747 ± 0.59	0.197
Male gender	221	9	0.550
Vaginal delivery	283	10	0.09
Apgar score at 5 min less than five	10	3	0.001
NICU admission	137	11	0.032
Fetal distress	73	9	0.009
Meconium stained amniotic fluid	28	2	0.012
Family history	15	1	0.022
Maternal age (years)	24.99 ± 3.3	25.35 ± 4.3	0.313

Maternal fever was present in only 1 % patients antenatally and they all had normal hearing, while rash was present in only 0.3 % of subjects and they also had no significant hearing loss. Blood transfusion history was present in total four patients and out of them three had normal hearing screening and remaining one had significant hearing loss. APH was present in 12 patients out of them ten had normal hearing and two had found significant hearing loss (Table 3).

**Table 3:** Distribution of cases according to antenatal history in relation to significant hearing loss

Antenatal history		Significant hearing loss			*χ* ^2^	*P*
		Absent	Present	Total		
Fever	No	383 (99.0 %)	18 (100.0 %)	401 (99.0 %)	0.188	0.883
	Yes	4 (1.0 %)	0	4 (1.0 %)		
Rash	No	386 (99.7 %)	18 (100.0 %)	404 (99.8 %)	0.047	0.956
	Yes	1 (0.3 %)	0	1 (0.2 %)		
Jaundice	No	383 (99.0 %)	17 (94.4 %)	400 (98.8 %)	2.844	0.089
	Yes	4 (1.0 %)	1 (5.6 %)	5 (1.2 %)		
Blood Transfusion	No	384 (99.2 %)	17 (94.4 %)	401 (99.0 %)	4.019	0.045
	Yes	3 (0.8 %)	1 (5.6 %)	4 (1.0 %)		
Leaking per-vaginum	No	375 (96.9 %)	17 (94.4 %)	392 (96.8 %)	0.334	0.564
	Yes	12 (3.1 %)	1 (5.6 %)	13 (3.2 %)		
Offending drug intake	No	385 (99.5 %)	18 (100.0 %)	403 (99.5 %)	0.093	0.954
	Yes	2 (0.5 %)	0	2 (0.5 %)		
Radiation exposure	No	387 (100 %)	18 (100.0 %)	405 (100.0 %)	-	-
	Yes	0	0	0		
Other	No	384 (99.2 %)	18 (100.0 %)	402 (99.3 %)	0.141	0.987
	Yes	3 (0.8 %)	0	3 (0.7 %)		
Drug addiction	No	382 (98.7 %)	17 (94.4 %)	399 (98.5 %)	2.142	0.143
	Yes	5 (1.3 %)	1 (5.6 %)	6 (1.5 %)		
Antenatal clinics attended	No	98 (25.3 %)	5 (27.8 %)	103 (25.4 %)	0.055	0.815
	Yes	289 (74.7 %)	13 (72.2 %)	302 (74.6 %)		
Maternal TORCH profile	No	386 (99.7 %)	18 (100.0 %)	404 (99.8 %)	0.047	0.829
	Yes	1 (0.3 %)	0	1 (0.2 %)		
Pre-eclampsia	No	374 (96.6 %)	18 (100.0 %)	392 (96.8 %)	0.625	0.429
	Yes	13 (3.4 %)	0	13 (3.2 %)		
Antepartum hemorrhage	No	377 (97.4 %)	16 (88.9 %)	393 (97.0 %)	4.350	0.037
	Yes	10 (2.6 %)	2 (11.1 %)	12 (3.0 %)		

The working cost for the institute for TEOAE was 250 Indian rupees (4 US dollars) and for AABR was 1300 Indian rupees (21 US dollars). The parents were not billed for either of the probes.

## Discussion

In our survey, among 415 of the total newborns screened 94.7 % (393) cases passed on TEOAE while 5.3 % [19] cases were referred for initial screening at birth.

Nagapoornima et al. conducted a similar study in India and screened a total of 1769 infants (1490: Not at risk; 279: At risk) & reported that 10 babies were having a hearing impairment [20]. The high incidence of hearing impairment seen in our study population could be explained because of neonatal population with different geographical area and also because of different maternal antenatal risk factors. There can be also some unseen environmental and genetic and epigenetic factors responsible for the high incidence of hearing impairment in our studies.

John et al. conducted study in Christian Medical College (C.M.C.) Vellore and evaluated 500 newborns and found 32 (6.4 %) neonates with negative response. These effects are quite comparable to our study. At the second stage screening on 32 (6.4 %) cases by Distortion Product Otoacoustic Emission DPOAE, eight (25 %) were extended to sustain a negative response [21] and similarly in our study four neonates had normal AABR in follow up.

Thomson et al. in their study of newborn screening, included 67,261 newborn from different states and 8.2 % mothers of babies were from 11–19 years age group while 23.1 % were from 20–25 years & 67.5 % mothers were a 25 + age group [22]. In our study case for maximum cases coming from 21–25 years age group was likely due to lower age for matrimony in our region as compared to the western world. We found no link between maternal age and hearing loss.

In subject done by John et al. [21], they reported that 5.2 % of cases of failed hearing screening by DOAE were from the birth weight less than 1500 g. Low birth weight is also included as a risk factor for hearing impairment in JCIH 2000 criteria [18]. A lower rate of hearing abnormalities in low birth weight in our subject population can be explained by either small sample size or high mortality in babies of low birth weight in our population. The screening should be a part of all neonates irrespective of weight, although the smallest, and tiniest neonates require AABR as a primary modality, but using only weight as a criteria is not advisable as full term neonates without any risk factors can also have hearing abnormalities.

Nagapoornima et al. [20] recorded that out of total eight cases screened with a family story of childhood sensorineural hearing loss two (25 %) cases were set up to cause hearing impairment. In our study, out of 18 cases of hearing loss, 94 % [17] cases had no family history of significant childhood hearing loss in any sibling of the child. Family history of hearing loss was present in 16 (3.8 %) children and out of these only one case had hearing handicap. This shows that although the family history is recognized as an independent risk factor for hearing loss but the neonates who don’t have any family history are also prone to have hearing abnormalities hence showing the importance of universal newborn screening of all neonates irrespective of family history of hearing loss.

John et al. [21] showed that a family history of hearing loss was present in seven cases out of 500 (1.4 %) [5]. This high level of hearing loss may be due to deficiency of awareness in our region & less access to health care services.

In our study, most of the antenatal risk factor for hearing loss were found non-significant except the history of antepartum hemorrhage and history of antenatal blood transfusion in the mother. The apparent cause of these antenatal risk factors associated with hearing loss may be due to intrauterine distress to fetus followed by birth asphyxia.

In our study, we concluded that the method of delivery does not play a significant role on newborn hearing impairment.

Out of the 18 neonates with significant hearing impairment nine cases where having the antenatal sign of fetal distress in the form of either meconium stained liquor, bradycardia, or tachycardia. When comparing these data, the difference was statistically significant (*p* < 0.01). This could be explained because signs of fetal distress like meconium stained liquor, bradycardia, or tachycardia indicates fetal hypoxia, which may lead to damage to cochlear cells and neuronal pathway leading to more significant hearing abnormality. These findings also strengthen the view that these neonates with perinatal asphyxia need to be screened for hearing assessment.

In the present study, 16.7 % [3] cases out of 18 cases of significant hearing loss were found to have Apgar score ≤5. This was strongly associated risk factor for neonatal hearing loss as fetal hypoxia can damage neonates hearing system and lead to AABR abnormality.

Similar outcomes were likewise received in the survey performed by Thomson et al. [21] on newborn screening. In his study 39 (1.2 %) babies of apgar ≤5 were found to cause failure of hearing screening. Low Apgar score is also included as a risk factor for hearing impairment in JCIH 2000 criteria [18]. So our study confirms it as associated risk factor.

According to JCIH 2000 criteria [18] an illness or condition requiring admission of 24 h or more to NICU has termed as a risk factor for hearing impairment. Our study confirms the same finding. This could be because the sick neonates are exposed to various ototoxic drugs or hypoxia, which increases the chances of hearing loss. The results also tell that although the sick neonates are more likely to have a higher incidence of hearing abnormalities, but the normal neonates with no postnatal sickness or risk factors can have hearing abnormalities, hence again highlighting the need for universal screening.

In our experience from a developing country, we encountered few difficulties for newborn screening. The availability of a trained audiologist and the cost are important issues. However, both OAE and AABR are paid by the institution in the majority of places, hence all parents from the low income and low middle income countries will not be able to get their newborn infant screened. As both OAE and AABR were free to our patients could be one of the reason for hundred percent follow up of all infants. All these problems can be overcome if the government makes hearing screening compulsory, and free of cost, and many more audiologists are trained for performing newborn screens. Though the initial phase of screening implementation may be costly because getting new screening machines, training new audiologist, and setting up new rehabilitation centers, but in the long term these interventions will be more cost effective and will help in reducing the burden on society because of hearing impairments.

In the nursery, the high risk neonates are more prone to suffer hearing problems. In the various studies done it has been shown that in these neonates AABR when compared with OAE has better sensitivity and specificity which highlights that in these neonates AABR should be a primary screening modality and in normal neonates OAE can be taken as a primary modality of screening [19, 23].

Hence the results of our study show that all neonates having a turbulent course in nursery and various risk factors, needs to be screened for hearing assessment and neonates with the abnormal OAE need to be followed up. Universal screening of hearing should be done as neonates with no risk factors can also have abnormal hearing as seen in our study and it can delay the diagnosis of hearing impairment and also intervention for rehabilitation.

The limitation of our study includes small sample size, and our inability to evaluate all the risk factors of JCIH criteria. The strong point of our study includes hundred percent follow up of all the newborns who had abnormal OAE and all these neonates had AABR done. This was possible as we educated all parents regarding the consequences of impaired hearingand maintained regular contact with them.

## Conclusion

Late identification of hearing loss presents a significant public health concern. However, without screening, children with hearing loss are usually not identified until 2 years of age, which results in significant delays in voice communication, language communication, social, cognitive, and emotional development. In contrast, early recognition, and intervention prior to 6 months of historic period has a significant positive impact on development.

Hence, there is an urgent need to incorporate universal neonatal hearing screening in all the neonatal health care facilities in India. While studying the facts like infrastructure limitations of our rural area where basic needs are deficient, there is a demand to employ cost-effective behavioural observation methods using calibrated noise making toys to screen all newborn infants.

Further prospective studies on a larger sample size are required to study the association of hearing impairment with various congenital syndromes and antenatal, perinatal, postnatal & demographic factors.
